# Diet, Lifestyle and Cardiovascular Diseases: Linking Pathophysiology to Cardioprotective Effects of Natural Bioactive Compounds

**DOI:** 10.3390/ijerph17072326

**Published:** 2020-03-30

**Authors:** Javad Sharifi-Rad, Célia F. Rodrigues, Farukh Sharopov, Anca Oana Docea, Aslı Can Karaca, Mehdi Sharifi-Rad, Derya Kahveci Karıncaoglu, Gözde Gülseren, Ezgi Şenol, Evren Demircan, Yasaman Taheri, Hafiz Ansar Rasul Suleria, Beraat Özçelik, Kadriye Nur Kasapoğlu, Mine Gültekin-Özgüven, Ceren Daşkaya-Dikmen, William C. Cho, Natália Martins, Daniela Calina

**Affiliations:** 1 Zabol Medicinal Plants Research Center, Zabol University of Medical Sciences, Zabol 61615-585, Iran; javad.sharifirad@gmail.com; 2 LEPABE—Department of Chemical Engineering, Faculty of Engineering, University of Porto, Rua Dr. Roberto Frias, s/n, 4200-465 Porto, Portugal; c.fortunae@gmail.com; 3 Department of Pharmaceutical Technology, Avicenna Tajik State Medical University, Rudaki 139, 734003 Dushanbe, Tajikistan; shfarukh@mail.ru; 4 Department of Toxicology, University of Medicine and Pharmacy of Craiova, 200349 Craiova, Romania; daoana00@gmail.com; 5 Department of Food Engineering, Faculty of Chemical and Metallurgical Engineering, Istanbul Technical University, Maslak, Istanbul 34469, Turkey; cankaraca@itu.edu.tr (A.C.K.); kahvecid@itu.edu.tr (D.K.K.); ozcelik@itu.edu.tr (B.O.); kasapogluk@itu.edu.tr (K.N.K.); gultekinmi@itu.edu.tr (M.G.-Ö.); 6 Department of Medical Parasitology, Faculty of Medicine, Kerman University of Medical Sciences, Kerman 7616913555, Iran; mehdi_sharifirad@yahoo.com; 7 Department of Food Engineering, Chemical and Metallurgical Faculty, Istanbul Technical University, Maslak Istanbul 34469, Turkey; gulsereng@itu.edu.tr (G.G.); esenol@firat.edu.tr (E.Ş.); evrendemircan@itu.edu.tr (E.D.); 8 Phytochemistry Research Center, Shahid Beheshti University of Medical Sciences, Tehran 1991953381, Iran; taaheri.yasaman@gmail.com; 9 Department of Agriculture and Food Systems, The University of Melbourne, Melbourne 3010, Australia; hafiz.suleria@unimelb.edu.au; 10 Bioactive Research & Innovation Food Manufac. Indust. Trade Ltd., Katar Street, Teknokent ARI-3, B110, Sarıyer, Istanbul 34467, Turkey; 11 Pladis TR R&D Department, Kısıklı mah., Ferah cad. Üsküdar İstanbul 34692, Turkey; ceren.dikmen@pladisglobal.com; 12 Department of Clinical Oncology, Queen Elizabeth Hospital, 30 Gascoigne Road, Hong Kong, China; 13 Faculty of Medicine, University of Porto, Alameda Professor Hernâni Monteiro, 4200-319 Porto, Portugal; 14 Institute for Research and Innovation in Health (i3S), University of Porto, 4200-135 Porto, Portugal; 15 Department of Clinical Pharmacy, University of Medicine and Pharmacy of Craiova, 200349 Craiova, Romania

**Keywords:** food, diet, lifestyle, classical and emerging risk factors, atherosclerosis, arterial hypertension, diabetes, coronary artery disease, plant-food bioactive compounds, cardio-protective pharmacotherapy

## Abstract

Heart and blood vessels disorders comprise one of the main causes of death worldwide. Pharmacologically active natural compounds have been used as a complementary therapy in cardiovascular disease around the world in a traditional way. Dietary, natural bioactive compounds, as well as healthy lifestyles, are considered to prevent coronary artery diseases. Pre-clinical and clinical studies reported that consumption of plant-food bioactive derivatives including polyphenolic compounds, peptides, oligosaccharides, vitamins, unsaturated fatty acids possess protective effects on cardiovascular diseases. This review aims to summarize the cardiovascular risk factors, pre-clinical studies and clinical trials related to cardioprotective properties of the plant-food-derived bioactive compounds. Molecular mechanisms by the natural bioactive compounds exert their cardiovascular protective properties have also been highlighted.

## 1. Introduction

The cardiovascular (CV) system provides blood circulation in the human body, which has the vital function of delivering oxygen and nutrients to the organs and tissues and removing carbon dioxide and other metabolic and waste products from the body [1]. Pathologies of the cardiovascular system include primary heart diseases, such as cardiomyopathy, and heart tumours. They also include heart tissue damage (e.g., infectious, infectious-allergic), dysmetabolic and systemic diseases and diseases of other organs [1,2]. 

Nowadays, cardiovascular diseases (CVDs) are one of the leading causes of death, ranking first globally [3]. According to the WHO, 17.5 million people die annually from CVDs, which accounted for 31% of all deaths in the world [3]. Approximately 7.4 million people die from coronary heart disease and 6.7 million people as a result of a stroke [4,5].

CV risk factors include e malnutrition, tobacco and alcohol use, stress obesity, sedentary lifestyle, hypertension, diabetes, hyperlipidemia and genetic predisposition [6]. Genetic baggage has an important contribution to the development of premature cardiovascular disease. A recent study showed that they sufferers are usually smokers, sedentary, hypertensive and dyslipidemic, but all of these factors can be modified. Five modifiable risk factors were evaluated: sedentary lifestyle, smoking, high blood pressure, diabetes, hypercholesterolemia. Almost three quarters of patients (73%) had at least three risk factors compared to 31% of healthy subjects. In both groups, the likelihood of developing coronary heart disease increased exponentially with each additional risk factor. The probability of illness was three, seven and 24 times higher in those with one, two and three or more risk factors. Genetic sequencing of all patients was performed and a genetic risk score for coronary heart disease was developed. Overall, the score was higher in the patients in the control group. The score was an independent predictor for premature coronary heart disease. However, the genetic contribution to the risk of coronary heart disease decreases as the modifiable risk factors decrease [7].

These data demonstrate the contribution of genetic predisposition to heart disease, but in patients with at least two cardiovascular risk factors the genetic cause plays a less decisive role in the development of the disease [8]. 

**Figure 1:**
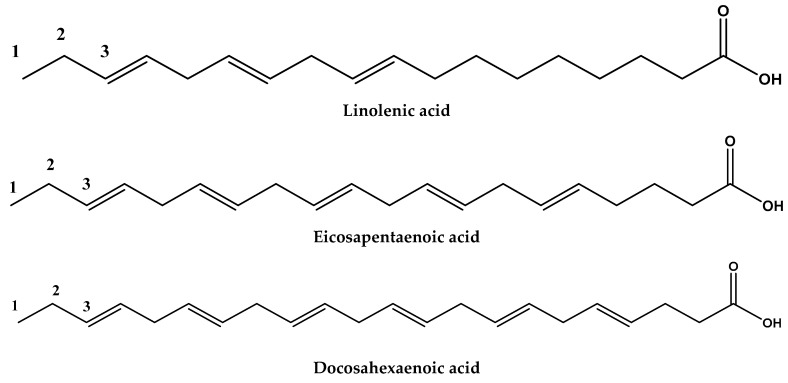
Chemical structures of some omega-3 fatty acids.

According to pre-clinical as also clinical studies, a correct diet has a cardioprotective effect on human health. CVDs can be prevented by consuming a diet rich in fruits, vegetables and low-fat dairy products, low in fat, red meat, sweets and sugary drinks, limiting soda and sodium chloride intake, and a sufficient intake of K^+^, Ca^2+^ and Mg^2+^, vitamin C and omega-3 fatty acids (Figure 1). These dietary lifestyles have a positive impact on the prevention of CVDs. Besides, restricting alcohol and tobacco consumption, weight control, daily exercise, ensuring healthy sleep, and avoiding stress are also recommended for the prevention of CVDs [9]. Plant-food bioactive molecules with different chemical structures, such as polyphenolic compounds, peptides, oligosaccharides, vitamins, fatty acids possess cardioprotective effects [10,11].

Among the multiple plant-derived bioactives, polyphenols are one of the most important groups of natural cardioprotective, antioxidant and anti-inflammatory agents found in human foods, including fruits, vegetables, grains, herbs, and seeds [12]. These molecules are secondary metabolites responsible for pigmentation, reproduction, growth, and protection against pathogens in plant-based foods [13,14]. Having a sugar residue linked to the carbon skeleton, the chemical structure of polyphenols differ solely in their additional linkages with other compounds [15]. Polyphenols can be basically divided into three sub-groups: flavonoids, non-flavonoids, and phenolic acids [16]. In the colon, polyphenols are initially digested into smaller phenolic structures by the gut microflora [17,18].

Polyphenols can improve cardiovascular health using inhibiting platelet aggregation, reducing vascular inflammation, modulating apoptotic processes, limiting low-density lipoprotein LDL oxidation and improving lipid profile [19].

Many studies have suggested that citrus fruits, cocoa-rich products and dark chocolates contain high concentrations of flavonoids related to CVD risk reduction [20,21]. Likewise, green tea and its extracts have a high level of flavan-3-ol molecules, epigallocatechin-3-gallate (EGCG), which are among important cardioprotective antioxidants. Black tea also has a powerful antioxidant capacity, but lower than green tea extract. Both teas/extracts help to decrease blood pressure in humans, which can positively affect the CV risk profile [20]. 

Resveratrol is a polyphenolic antioxidant found in plants, such as grapes, blackberries, tomatoes, red currants, and blueberries. Resveratrol stimulates the production of the sirtuin-1 (SIRT 1) protein called the “longevity gene” and thus helps maintain cellular health, slowing the ageing process. Resveratrol is considered a key ingredient in prolonging the lifespan [22]. Resveratrol protects the cardiovascular system due to its anti-inflammatory properties, it reduces the risk of atherosclerosis, reduces platelet aggregation and myocardial fibrosis.

Carotenoids are valuable liposoluble molecules. They have pro-vitamin A activity and have anti-oxidant potential, decreasing the risk of several chronic diseases, such as cancer or CV diseases, macular degeneration and age-associated biological transformations. 

Omega-3 fatty acids, including omega-3 docosahexaenoic acid (DHA) and eicosatetraenoic acid (EPA), are responsible for normal brain development, normal vision and a lower CV disease risk [23]. EPA and DHA have anti-inflammatory and cardioprotective effects, including anti-arrhythmic, anti-thrombotic properties. They also trigger a decrease in blood pressure, strengthen the endothelial function and diminish the growth of atherosclerotic plaque [24], so they seem to be promising biomolecules with antihypertensive, antioxidant, anti-depressive, antiaging and antarthritic effects [25]. 

The present review summarizes CV risk factors, pre-clinical and clinical trial related to plant-food-derived bioactive compounds in CVDs. 

## 2. Cardiovascular Risk Factors, Diet, Lifestyle and Cardioprotective Benefits of Plant-Food Derived Bioactive Compounds 

### 2.1. Sedentary Lifestyle

**Figure 2:**
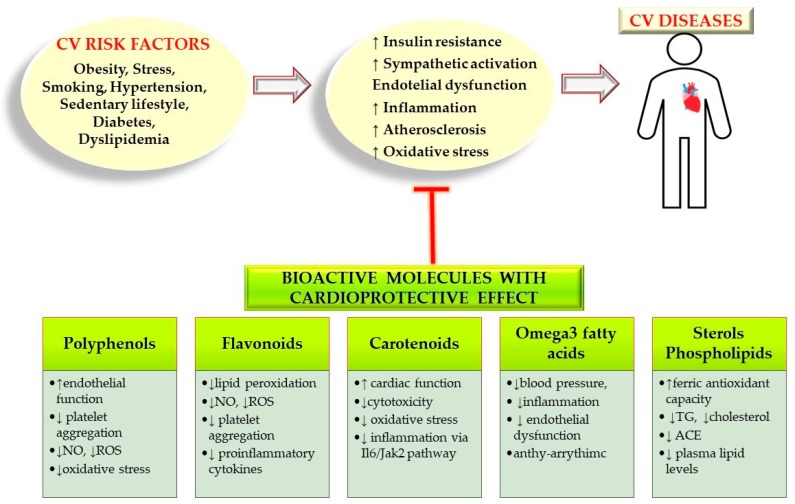
Schematic overview of CV risk factors, pathophysiology of CV diseases and the cardioprotective mechanisms and effects of plant-food derived bioactive compounds. *Abbreviations*: Cardiovascular (CV), Nitric oxide (NO), Reactive oxygen species (ROS), Interleukin-6 (IL-6), Janus tyrosine kinase 2(JAK2), Triglycerides (TG), Angiotensin-converting enzyme (ACE).

The incidence of chronic diseases, especially CV disease, have markedly raised in the last century [26]. A sedentary lifestyle increases insulin resistance, induces obesity, increases blood glucose levels, plasma lipids and prothrombotic factors. According to a recent report, 1/3 of worldwide deaths are due to CV disease [27] (Figure 2).

Several researchers have investigated the relationship between sedentary behaviour and CV disease. Warren et al. [28] revealed that men who spent >10 h in a car/week had 82% greater risk of mortality by CV disease compared those who spent <4 h per week. Young et al. conducted a study on 82,695 men aged ≥45 years and found that the hazard ratio of heart failure increased as the physical activity decreased [29].

According to a report from the American Heart Association, sedentary behaviour is linked with increased CV-specific and overall mortality and regarded as a disease burden. Besides, different studies have also shown that higher sedentary time is related to depressive symptoms and decreased psychological well-being and health-related quality of life [30]. Additionally, a study with 10,261 adults found that individuals who have light, moderate or vigorous physical activity had a significantly lower risk for CV disease mortality, regardless of their metabolic risk factors [31]. Gobbo et al. concluded that the adherence to a few modifiable risk factors, such as physical activity, moderate alcohol consumption, not smoking, and avoiding obesity, reduced the risk of incident heart failure in 50% cases. [32].

### 2.2. Hypertension

Arterial hypertension and endothelial growth factor-linked polymorphisms are reported to contribute to vascular damage [33,34]. Nicoll et al. studied 15,769 patients on the relationships between conventional CV risk factors (age, gender, ethnicity, DM, dyslipidemia, hypertension, obesity, exercise) and coronary artery calcification, and found that hypertension and diabetes have the strongest association with coronary artery calcification [35].

Several plant-food bioactives being identified as potentially useful on both prevention and reduction of some highly prevalent CV risk factors (Figure 2). Berberine, green tea, cocoa, lycopene, aged garlic extract, pycnogenol, resveratrol, grape seed extract, beetroot juice, olive oil and ascorbic acid are among the plant-derived nutraceuticals with promising blood pressure lowering-activity [36]. In a comprehensive study of several plant-derived extracts, these were able to lower blood pressure [37]. Resveratrol lowers blood pressure due to its antioxidant properties. It helps in the production of nitric oxide in the body which causes arterial vasodilation and thus decreases blood pressure.

Angiotensin-converting enzyme (ACE) has been known to play a crucial role in arterial blood pressure regulation and CV function [38]. Therefore, the inhibition of this enzyme via plant-food bioactives is a significant area of research in the treat hypertension [39,40]. Numerous studies have reported plant-based ACE-inhibitors from beans, cereals, legumes, nuts, seeds and other sources [41,42].

Phenolic and flavonoid contents of fruits have been significantly correlated with their respective ACE inhibitory effects [43]. Fruits seeds are cheap and sustainable sources for bioactive constituents. Apricot and olive seed protein hydrolysates have been documented to reduce micellar cholesterol solubility with a high antioxidant and ACE inhibitor capacities [44]. Vicilin, a major storage protein of a nutritive legume, namely mung bean, also has good antioxidative potential and ACE inhibitory activity [45].

### 2.3. Smoking

In addition to hypertension, smoking considered as one of the key factors increasing the risk of vascular diseases [33]. Tobacco smoke contains condensed tar particles with oxidizing and pro-oxidant effects that produce free radicals, thus increasing lipid peroxidation in biological membranes. Free radicals and oxidative stress play a crucial role in a broad spectrum of CV disease pathophysiologies [46]. Smoking also plays a vital role in atherogenesis, coronary spasms, coagulation mechanism disorders, increased oxidation of LDL-cholesterol, platelet aggregation, fibrinogen growth, diseases of endothelial function, and altered lipid profiles (reduced HDL cholesterol). Smoking also can produce DNA mutations in lung cells that increase the risk of lung cancer.

Recent studies indicate a relationship between major depression and CVDs, implying that both diseases may share common physiological pathways which also include lifestyle factors such as physical activity and smoking behaviour [47]. It has been reported that most depressed patients have high rates of adverse health behaviours, including smoking [48].

Several plant-based antioxidant agents, such as aged garlic extract, *Angelica giga,* celery, *Artemisiae annuae herba*, oat extracts, cinnamon, soy extracts, hibiscus, flaxseed, wolfberry, lycopene, basil, sesamol, cocoa and ginger none are among the plant-derived bioactives that have shown to be useful in reducing atherosclerosis induced by smoking [49,50]. Other natural compounds like flavonoids, polyphenols, derivatives of polyphenolcarboxylic acids, hydroquinone or naphthoquinone and tannins have antioxidant effects.

The proanthocyanins from bilberries (*Vaccinium myrtillus*) have anti-inflammatory effects by inhibiting the release of pro-inflammatory cytokines and chemokines (α-TNF, IL-1, IL-6, IL-8). Other natural compounds such us caffeic acid, gallic acid, chlorogenic acid, cvercetin derivatives have antioxidant and antihistamine effects [51].

The extract from the rosehip (*Rosa canina*) has antioxidant action due to its high content of vitamin C and polyphenols [52]. The extract from *Betula pubescens* buds is rich in polyphenols, and flavonoids such as hyperoside and quercetin, caffeic acid derivatives and betulinic acid, which support antioxidant action [53].

Recent studies showed that grape seed extract is one of the most effective antioxidants due to its phytocomplex of *trans*-resveratrol and oligomeric proanthocyanins [54]. It also has a vasoprotective and cardioprotective effect due to its ability to neutralize free radicals, reduce atherosclerosis and ameliorate smoking-induced venous disturbances [55].

### 2.4. Stress

Stress has also been linked to CV disease risk [56]. Post-traumatic stress disorder and chronic stress have been connected to CV disease risk as they can affect the body’s ability to regulate its stress-response system resulting in raised heart rate, blood pressure and atherosclerosis [57].

Three pathophysiological mechanisms of chronic cardiovascular disease are correlated with stress: i) disturbance of the hypothalamic-pituitary-adrenal axis, by increasing the level of serum cortisol due to stress, ii) inflammation of the arterial (endothelial) wall, which causes atherosclerosis, iii) increasing the tone of the sympathetic vegetative nervous system. X

Several studies indicate that individuals with post-traumatic stress and chronic stress have an increased risk of hypertension, hyperlipidemia, obesity, and CV disease [58].

Increased plant-based foods consumption has been linked with promoting health and well-being by reducing the CV disease risk, as they are rich sources of fiber, essential micronutrients such as vitamins and minerals and contain a wide group of bioactive compounds including flavonoids, phenolic acids, carotenoids, and plant sterols [59].

Runnie et al. investigated the vasodilatory actions of nine edible tropical plant extracts. They reported that sweet potato, betel, cashew, maman, papaya, and mint leaves extracts showed >50% relaxing effect on aortic ring preparations [60].

L-Theanine is known to be responsible for a sense of relaxation upon consumption of green tea [61]. Steptoe et al. [62] reported that 6 weeks of tea consumption resulted in lower post-stress cortisol and greater subjective relaxation, together with reduced platelet activation compared with placebo.

Numerous studies have shown that St. John’s wort (*Hypericum perforatum)* extract is the most potent natural antidepressant. It is recommended for states of fear, tension and stress. Due to the hyperforin content, a natural derivative with sedative properties is especially useful in depressive state. It can increase the amount of serotonin produced in the brain; an effect similar to antidepressant drugs [8].

Ginseng also has anti-stress action, reducing physical and mental fatigue, increasing the power of concentration, memory and work performance. It increases muscle activity and improves the metabolic activity in the brain [63].

### 2.5. Obesity

Obesity has become a global concern known as “globesity”. According to WHO, about 13% of the world’s adult population was obese in 2016 [64]. Obesity is not only an imbalance between calories consumed and energy expended but rather a complex condition with risk factors, including genetic and behavioural factors [65].

Cardiovascular disease in obesity is linked to all the mechanisms of vascular disease: dyslipidemia, hypercoagulability, platelet dysfunction, insulin resistance and type 2 diabetes, inflammation. Pathogenic pathways of vascular disease are interconnected by circulating factors, and adipose tissue is an important source for many circulating mediators that promote insulin resistance, pro-inflammatory and prothrombotic status, and thus promote cardiovascular disease [66].

Several chronic diseases linked to obesity, including CVDs [33,67] type II diabetes [68], hypertension [69], musculoskeletal disorders [70], and certain types of cancer [71]. Current strategies to prevent and/or treat obesity involve lifestyle interventions, pharmacotherapy and bariatric surgery, which have extensively been reviewed recently [65].

Plant-derived bioactive compounds are considered as an emerging tool to develop anti-obesity strategies, where a significant pool of evidence has already been collected. [72]. Among the phytochemicals studied, a special attention has been paid on phenolic compounds from a variety of sources [73,74], such as tea [75,76], grapes [77], ginseng [78], red to purple colour fruits [79,80,81], cruciferous vegetables [82,83], etc. Several bioactive compounds found in oils, such as tocopherols and tocotrienols, phenolic acids, flavonoids, and sterols have anti-obesity effects [84].

Resveratrol can prevent fat deposits, help regulate insulin levels, reduce body weight. This fact is supported by studies that show that SIRT 1 protein, activated by resveratrol, has the ability to protect the body against obesity.

Many studies have linked consumption of mono- and polyunsaturated fatty acids, including conjugated linoleic acid, instead of their saturated counterparts, with reduced body weight [85]. Bioactive peptides from soybean have led to promising results for obesity treatment [86]. Positive effects of sulphur-containing components of onion on obesity prevention have been reported [87]. Pulses, such as dried beans, dried peas, chickpeas, and lentils, are nutrient-dense and yet low in fat, calories, and glycemic index foods; therefore, they are considered as one of the specific foods that could help reducing obesity with promising results [88].

Starting from 1935, when McCay et al. showed for the first time that the lifespan of rats can be extended by decreasing food intake [89], the impact of caloric restriction (CR) in reversing the ageing process and the effects of different pathologies started to be more and more studied. The aging process was retarded by long-term CR in different organism models, from yeast to short-lived mammals as mice and rats [90]. There are several factors that influence the efficacy of CR as the total duration and the moment of life when the regiment is initiated. There are several types of CR except from life-long CR that is very hard to keep in the modern society, as short-term CR (3 months), overnight starvation or prolonged starvation (48 h). The Comprehensive Assessment of the Long-term Effects of Reducing Intake of Energy (CALERIE) trials that investigated the effects of CR in healthy, non-obese human beings showed that short-term CR (6-12 months) decreased the fasting insulin levels, body temperature and improved the major risk factors for cardiac diseases [91], while long-term CR (2 years) improved quality of life, sleep and sexual functions without negative effects [92]. Another clinical trial that investigated the effect of 6 months CR in patients with type 2 diabetes with abdominal obesity, showed that the CR improved glomerular hyperfiltration, decreased cardiovascular risk factors and improved insulin sensitivity [93]. The cellular mechanisms through which CR provides anti-aging and cardioprotection are diverse, and include influencing oxidative stress, inflammation, apoptosis and authophagy [94]. Although CR has shown its beneficial effects in aging and cardioprotection, many persons cannot adapt to long term CR, so other alternatives to mimic the effects of CR have been investigated and a new class of chemicals known as CR mimetics has appeared. The chemicals from this category are defined as any agent or intervention that can reproduce the effects of CR without requiring the decrease in food intake [95]. One of the cellular mechanisms implicated in the beneficial effects of CV is induction of autophagy determined by depletion of intracellular acetyl coenzyme A associated with deacetylation of cellular proteins [96]. Several natural compounds have shown CR mimetic effects by inducing autophagy such as curcumin [97], garcinol [98], anacardic acid [99], epigallocatechin-3-gallate [100] and resveratrol [101]. A study that compared resveratrol supplementation and long-term CR showed that resveratrol can modify the heart like long-term CR [102].

### 2.6. Diabetes

Together with the obesity epidemic, type II diabetes (T2D), which is a result of the body’s ineffective use of insulin due to inadequate insulin secretion by pancreatic β cells, has become a public health challenge in many countries. According to the WHO’s Global Report, 422 million people worldwide (adult population, 8.5%) had diabetes in 2014 [103].

Increased plasma glucose levels is responsible for glycosylation of proteins in the arterial walls and nerves and causes alteration of their structure, thus decreasing their resistance to the action of oxygen free radicals, increasing inflammation at the endothelial level, with the deposition of VLDL and LDL cholesterol particles at this level and the formation of atheroma plaque.

The combination of a sedentary lifestyle, unhealthy diets, overweight/obesity, smoking, and excessive alcohol intake are presumed responsible for 90% of T2D cases [104]. Furthermore, T2D reported being among the leading causes of blindness and lower limb amputation, in addition to being a significant risk factor for CVDs [105]. Its close relationship with obesity makes T2D effectively controlled by changes in dietary habits [106].

The Mediterranean diet, characterized by foods with rich polyphenol content such as olive oil, nuts, and red wine, has been linked to a reduced risk of T2D [107]. The role of phenolic compounds in T2D treatment has been extensively reviewed recently [108,109].

Several phytochemicals, including alkaloids, polyphenols, flavonoids, terpenoids, saponins and lignans were reported to target genes so that the consumption of foods with these compound would have anti-diabetic properties [110]. Coffee and tea consumption, probably due to the phenolic compound content, was inversely related to T2D risk [111]. Saffron, with its high β-carotene content, has hypoglycemic effects [112]. Prebiotics such as arabinoxylan, β-glucan [113] and dietary fibre [114] from several cereals improve glucose metabolism.

Peptides derived from soybean, especially those in fermented soybean products, were shown to prevent T2D onset [86]. Pulse ingredients [115] and whole grain [116] have also been recognized for their role in preventing or managing diabetes.

### 2.7. Dyslipidemia

Dyslipidemia is the condition of having an abnormal amount of lipids (triglycerides, cholesterol, fat phospholipids) in the blood. More specifically, it refers to increased total cholesterol (TC), low-density lipoprotein cholesterol (LDLc) and triglyceride (TG) levels, as well as low levels of high-density lipoprotein cholesterol (HDLc).

In dyslipidemias, hepatic LDL receptors become incompetent; LDL circulates more through the body, its degree of oxidation increases and contact with the vascular endothelium is prolonged. Thus, the atherogenic effect is increased [117]. Atherosclerosis derived from dyslipidemia is characterized by deposits of atheroma plaques on the internal walls of medium and large arteries, with thickening of the arterial wall and loss of elasticity. Atherosclerosis is the leading cause of mortality globally, as it is the leading cause of fatal cardiovascular events [118].

WHO reported that 1/3 of ischemic heart disease is due to high TC levels [119]. A reduction of 5–6% in serum low-density lipoprotein cholesterol (LDLc) decreases the CVDs risk by 7–12% [120], and a 3% increase in high-density lipoprotein cholesterol (HDLc) lower the risk by 6–9% [121].

Since lifestyle modifications do not cause a significant improvement in lipid profile, and the use of drugs can have adverse side effects (especially for low-risk patients), non-pharmacological alternatives for dyslipidemia control is gaining attention.

Phenolic compounds have been linked to a positive effect on dyslipidemia [122,123]. Those from cocoa have provided consistently positive results [124]. Tea, being the most popular soft drink worldwide, and far more commonly than wine, also contains polyphenols, which possess antioxidant activity together with vasorelaxant effect, protective effect against endothelial dysfunction and hypolipidemic effect [125].

Flavonol supplementation significantly reduced total cholesterol (TC), LDLc and triglyceride (TG) levels [124]. Several other phytochemical combinations with proven positive effects on dyslipidemia treatment have been reported [126]. Among those, red yeast rice extract rich in monacolins, derived by fermentation of rice, has gained special interest. Phytosterols have been reported to effectively reduce LDLc [127]. Soybean components, both proteins and isoflavones, reduce LDLc while increasing HDLc [128].

Summarized data related to CV risk factors, lifestyle, pathophysiological data and cardioprotective properties of plant-food derived bioactive compounds are shown in Table 1.

## 3. Cardioprotective Properties of Plant-Food Bioactives—Preclinical Studies

CV diseases are the leading cause of death globally [129]. The importance of diet in the increasing prevalence of CV risk factors, including serum lipid levels, glucose levels, hypertension, obesity, endothelial dysfunction, inflammation and/or oxidative stress been well established in the literature [130,131,132] (Table 2).

### 3.1. In Vitro Studies

For the evaluation of bioactives and/or food extracts in terms of the pharmacological activity, in vitro measurements using chemical and enzymatic reactions that occur in glass tubes, cuvettes and microplates are conducted in the first place [133]. There is a promising evidence related to the bioactive antioxidant potential through in vitro studies. [134]. In this regard, inhibition of critical enzymes, or other cellular mechanisms, including antioxidant, anti-inflammatory, hypo-cholesterolemia, hypolipidemic activities are assessed by taking advantage of several substrates, media, and analytical techniques [135].

A large number of molecules with distinct chemical structures, including polyphenolic compounds, peptides, oligosaccharides, vitamins, and fatty acids, have been reported as having cardioprotective activity [136,137].

Thus, long-chain omega-3 polyunsaturated fatty acids (LCn-3PUFA) have been among the most evident bioactive compounds possessing beneficial cardioprotective effects during the last decade. They include the plant-derived α-linolenic acid (ALA, 18:3*n*-3), and the fish-oil-derived EPA (20:5*n*-3) and DHA (22:6*n*-3) [138,139,140,141]. The cardiovascular protection conferred by polyunsaturated omega-3 acids is due to their actions on the metabolism of lipids, vessels and platelets, through which they achieve an anti-arrhythmic effect, lowering blood pressure, reducing inflammation and improving endothelial dysfunction, increasing autonomic vascular tone, reducing platelet aggregation and stabilizing the atheroma plate [142].

In vitro studies showed that LCn-3PUFA improved endothelial function [143]. Although a fall in mortality from CVDs, most deaths are caused by sudden cardiac deaths associated with fatal arrhythmia [144]. The antiarrhythmic actions of LCn-3PUFA have been shown in vitro using cultured neonatal cardiac myocytes. Several mechanisms have been proposed for the antiarrhythmic potential of LCn-3PUFA, such as cell membrane structure modification, direct effect on calcium channels and cardiomyocytes and their role in eicosanoid metabolism [145].

**Figure 3:**
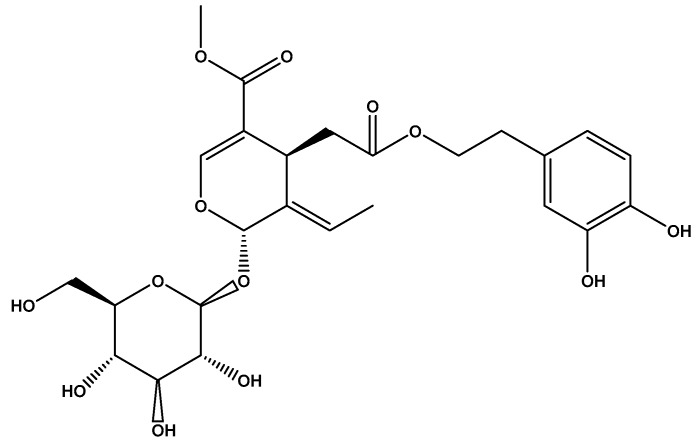
The structure of oleuropein.

Oleuropein and hydroxytyrosol, the most abundant polyphenols present in olive oil, has also been indicated as cardioprotective agents, due to strong radical scavenging properties in several experimental models [146]. The main glycoside in olives that is also responsible for the bitter taste, oleuropein, is the ester of oleanolic acid and 3,4-dihydroxy phenyl ethanol (Figure 3) [147]. The in vitro effects are associated with extracellular hydrogen peroxide production [148]. 

The ability of oleuropein to inhibit prooxidative processes has been shown in vitro on human LDL and on Caco-2 cells [149]. A large body of research has supported the in vitro cardioprotective potential of berry fruits rich in flavonoids, through improvements in plasma lipids and vascular function as well as systemic and vascular inflammatory response reduction [150,151]. LDL oxidation inhibition and induction of LDL receptor expression in hepatocytes have been demonstrated by cranberries consumption [152]. In addition, extracts from several berries, including raspberries, blueberries, blackberries and muscadine grapes inhibit in vitro expression of matrix metalloproteinase 2 (MMP-2) and matrix metalloproteinase 9 (MMP-9) [153,154].

Anthocyanins, a large sub-group of flavonoids, are water-soluble plant pigments that confer the red, purple, and blue colour to fruits, flowers, and leaves [155,156]. Their cardioprotective effects have been claimed as being mostly located at the endothelial cell level, contributing to vascular homeostasis [157]. Tenore et al. demonstrated that low doses of lyophilized wine made from Aglianico red grape (a black grape is grown in Italy) confer protection against physiological ROS and doxorubicin-induced oxidative injury in H9C2 rat cardiomyocytes cell lines. [158].

The cardioprotective effect of aspalathin (flavonoid of rooibos tea) in H9C2 cardiomyocytes has been linked to reverse in metabolic abnormalities by activating adiponectin (ADIPOQ) gene. At the same time, modulates peroxisome proliferator-activated receptor γ (Pparγ) and element-binding transcription factor 1 (Srebf1/2) expression and reduces inflammation via the Interleukin-6/ Janus kinase 2 (Il6/Jak2) pathway. Together with increased B-cell lymphoma 2 (Bcl-2) expression, all these effects prevent myocardium apoptosis [159].

The efficacy of cinnamic acid (a phenolic acid present in cinnamon), indicated an improved mitochondrial function in vitro and prevented apoptosis in H9c2 cardiomyocytes [160].

Dealcoholized red wine and cacao procyanidin trimers/pentamers can lead to in vitro increase of PAC-1 binding and P-selectin expression in blood and showed to modulate platelet activation, a biomarker of CVDs [161].

Cocoa flavanols have been reported to decrease vascular arginase activity in human endothelial cells [162]. Flavan-3-ols and procyanidins have shown ACE inhibitory activity, with the effect being dependent on the number of epicatechin units forming the procyanidin [163]. A two-stage alcalase–protamex hydrolysis of almond protein resulted in two ACE inhibitory peptides on the endothelial function of human umbilical vascular endothelial cells [164].

### 3.2. In Vivo Studies

The effects of bioactive compounds are generally assessed in vivo studies by the measurement of systolic blood pressure (SBP) of the animal model and analysis of the variation on mean arterial pressure to determine the chronic and acute effect of a compound, respectively [165,166,167,168]. In addition to the combination of bioactive compounds, the definition of the individual compounds’ mechanism of action has also been carried out by in vivo studies. [134]

The ACE-inhibitory activities of bioactive compounds can be assessed by periodic blood pressure measurements in spontaneously hypertensive rats (SHRs), after intravenous (i.v.) or intraperitoneally (i.p.) injection and oral gavage [41,42,169]. Hemodynamic and arterial baroreflex are evaluated due to the fact that baroreflex mechanisms may be effective on the long-term control of sympathetic activity and blood pressure [170,171].

Apart from these approaches, the antioxidant activity of a compound can be investigated due to the prevention of oxidative stress by the bioactive compound and their beneficial effect on CVDs [134,172].

Delivery of bioactive compounds to the target organ has also been considered as another parameter to determine the effect of the compound on the health [173,174]. Albumins were shown to bind n-3 PUFAs and to cause a greater reduction on their free form concentrations [175]. In this sense, the determination of the transfer of the bioactive compound to the heart is essential for CV health and could explain through the measurement of heart rate variability [176]. Several studies are reporting the activity of plant-based bioactive compounds on CV health. [10,11,177].

In this sense, [178] in a recent study have been investigated the effect of sterols, tocopherols, phospholipids, phenols, coenzyme (Co) Q9 and CoQ10 on plasma lipids and antioxidant defenses. Rats were fed with rapeseed oil at 20% of level and plasma and liver samples were evaluated. Depending on the administration of micronutrient, the triglycerides and cholesterol levels were reduced. Indeed, the ferric antioxidant capacity, ferric antioxidant capacity significantly increased, and lipid peroxidation decreased.

As similar, Ronchi et al. have assessed the antihypertensive effect of phytochemicals obtained from mango leaves by investigating in vivo ACE inhibitory activity of the extracts. Spontaneously hypertensive rats (SHRs) and Wistar rats were feed with enalapril as a positive control, a dichloromethanic fraction of mango leaves (100 mg/kg; twice a day) or a vehicle control for 30 days. Hemodynamic and arterial baroreflex assessment of the dichloromethane fraction of mango leaves has been performed, as well. In addition to inhibition of ACE, cardiac hypertrophy in spontaneously hypertensive rats treated with the dichloromethane fraction decreased and the ratio heart weight/body weight increased [166].

Zarei et al. have investigated in a recent study the antihypertensive activity of peptides obtained from palm kernel. Protein hydrolysates from palm kernel administered to rats (150 mg/kg, 75 mg/kg and 37.5 mg/kg). Different doses of hydrolysate concentration resulted in various SBP level and the highest modulating activity in rats was observed at 75 mg/kg hydrolysate [179].

*In vivo* antioxidative stress effects of polyphenolic compounds in pomegranate peel were also investigated. Oxidatively stressed were administrated ellagic acid, punicalin, and punicalagin at the concentration of 10 mg/kg for 21 days [180]. Total antioxidant capacity, superoxide dismutase (SOD), glutathione peroxidase (GPx) and glutathione (GSH) activity and malonaldehyde content were determined in plasma, liver and intestine of examined rats through the experiment. Ellagic acid distinguished thanks to its in vivo antioxidative activity against oxidative injury, especially for intestinal damage.

The main problem in natural compounds research is the way of translating the results of in vitro and in vivo studies into therapeutics. The main problems are related with the bioavailability and metabolism of natural compounds in humans, information that many times are missing. Taking as example, resveratrol, one of the most studied natural compounds lately, we saw that its low bioavailability and fast metabolism make difficult to estimate the correct dose for the targeted effect [181]. Studies have been shown that the cardiac protective effects of resveratrol observed at doses between 2.5 and 5 mg/kg/day were reversed when the doses were increased to doses of 25–50 mg/kg/day [182]. Another problem in translating the results from animals to humans is associated with safety and efficacy in humans that definitely needs the development of clinical safety trials and evaluation of potentially drug interactions that can have negative effects on the individual. We can conclude that even though there is a huge amount of preclinical studies that have shown the beneficial effects of several natural compounds, these studies should be better designed in order to try to simulate as much as possible the real-life scenario of human pathologies in order to assess and facilitate translation into the clinic.

## 4. Clinical Trials of Natural Bioactive Molecules with Cardioprotective Properties

According to epidemiological studies, consumption of fruits, vegetables, olive oil, wine, legumes and whole-grain included in the Mediterranean diet has a cardioprotective effect on human health [183]. Fruits and vegetable consumption reduces the CV disease risk [184] at various levels. The cardioprotective effects of foods rich in polyphenols (mainly anthocyanins) [185] were shown to improve endothelial function and plasma lipid profiles, inhibiting abnormal platelet aggregation and reducing inflammation [186]. Protection against oxidative stress is the principal action of anthocyanins to prevent CVDs [187]. The endothelium is a barrier between the blood and vessel wall, that controls vascular function, by responding to various hormones, neurotransmitters and vasoactive factors. If the atheroprotective balanced production of vasoactive factors is disrupted, this leads to endothelial dysfunction. 

**Table 2:** “*In vitro*” and “*in vivo*” preclinical studies - cardioprotective effects and molecular mechanisms of plant-food bioactives.

Natural Compound	Model	Molecular Mechanism of Action	Ref
***In Vitro* Preclinical Studies**			
omega-3 polyunsaturated fatty acids	neonatal cardiac myocytes	↑endothelial function antiarrhythmic potential: cell membrane structure modification, direct effect on calcium channels and cardiomyocytes, role in eicosanoid metabolism	[143]
oleuropein	human LDL and Caco-2 cell lines	↓prooxidative processes	[149]
flavonoids	hepatocytes cell lines	↓plasma lipids, ↑vascular inflammatory response reduction ↓LDL oxidation, ↑LDL receptor expression, ↓ MMP-2, ↓MMP-9	[150]
anthocyanins	H9C2 cardiomyocytes cell lines	cardioprotective effects on endothelial cell level, ↑vascular homeostasis ↓ROS	[155] [157] [158]
aspalathin (flavonoid from rooibos tea)	H9C2 cardiomyocytes cell lines	reverse in metabolic abnormalities by activating ADIPOQ gene, while modulates Pparγ and SREBF 1/2 expression, ↓inflammation via Il6/Jak2 pathway ↑Bcl2 expression, ↓myocardium apoptosis	[159].
cinnamic acid (phenol from cinnamon)	H9C2 cardiomyocytes cell lines	↑mitochondrial function ↓apoptosis	[160]
dealcoholized red wine and cacao procyanidin trimers/pentamers	*ex vivo* platelet activation	↑PAC-1 binding, ↑P-selectin expression in blood ↓platelet activation	[161]
cocoa flavan-3-ols	human endothelial cells	↓vascular arginase activity ↑ACE inhibitory activity	[162] [163]
almond protein	human umbilical vascular endothelial cells	↑ACE inhibitory peptides, antihypertensive effect	[164]
***In vivo* preclinical studies**			
sterols, tocopherols, phospholipids, phenols, coenzyme (Co) Q9 and Q10	rats	↓triglycerides, ↓cholesterol ↑ferric antioxidant capacity, ↑ SOD, ↑GPx, ↑GSH, ↓lipid peroxidation	[178]
dichloromethanic fraction of mango leaves	spontaneously hypertensive rats	antihypertensive effect ↓cardiac hypertrophy in spontaneously hypertensive rats treated with the dichloromethanic fraction, ↑ ratio heart weight/body weight	[166]
polyphenols (from pomegranate peel)	rats	↑antioxidative activity against oxidative injury (especially for intestinal injury)	[180]

*Abbreviations*: low-density lipoprotein (LDL), matrix metalloproteinase2 (MMP-2), matrix metalloproteinase 9 (MMP-9), peroxisome proliferator-activated receptor γ (Pparγ), sterol regulatory element-binding transcription factor 1 (SREBF 1/2) expression, reactive oxygen species (ROS), procaspase-activating compound 1 (PAC-1), superoxide dismutase (SOD), glutathione peroxidase (GPx), glutathione (GSH).

NO is a vasoactive factor released by the endothelium [188]. NO influences the development of atherosclerosis and many aspects of inflammation, hence, NO, inflammation, and endothelial dysfunction are biomarkers of CVDs risk [187].

In an observational study, the relation of total polyphenol intake and the risk of CV events within three groups consuming a Mediterranean diet was assessed. The authors found an inverse association between total polyphenol intake (particularly lignans, flavanols, and hydroxybenzoic acids) and CV disease risk [189].

A single-blind study for hypertensive men was employed by Asgary et al. who demonstrated the acute effects of pomegranate juice consumption on blood pressure and markers of endothelial function. This fruit is known to be rich in anthocyanins, catechins, quercetin, rutin and other phytochemicals (e.g., ellagitannins), and antioxidant vitamins, exhibiting antioxidant, anti-inflammatory and cardioprotective properties. The juice showed promising acute hypotensive properties. Due to the radical-scavenging effect of anthocyanins and hydrolysable tannins, pomegranate juice exhibited antioxidant activity. Besides, it also lowered blood pressure due to its ACE activity [190].

In a placebo-controlled crossover study [191], the effects of cranberry juice (rich in anthocyanins and other polyphenolic compounds) consumption on vascular function in patients with coronary artery disease were investigated. Chronic cranberry juice consumption reduced carotid femoral pulse wave velocity (a clinical measure of arterial stiffness). Also, it influenced the stiffness of the central aorta, which is a measure of vascular function. Yet, the authors only determined an acute benefit but not a chronic effect on endothelial vasodilator function.

The impact of high intakes of relatively pure anthocyanins on blood pressure determined in a clinical study [132]. In contrast to previous studies, they concluded that anthocyanins did not exhibit anti-hypertensive activity in borderline hypertensive men. The authors suggested that other substances other than anthocyanins might show antihypertensive effects.

Stull et al. reported in a double-blind and placebo-controlled study that daily dietary consumption of blueberries did not affect blood pressure and insulin sensitivity, but increased endothelial function (reactive hyperemia index) over 6 weeks in subjects with metabolic syndrome (blueberry, *n* = 23; and placebo, *n* = 21) [184].

In a randomized clinical trial, moderate alcohol consumption, especially red wine, lowered the risk of CVDs. CV mortality rate is lower in France than other countries consuming similar saturated fats due to red wine intake, called “French paradox” [192]. It suggested that there is an interaction between lipoprotein(a) and other CVDs risk factors (LDL, HDL, homocysteine) [193]. The ethanolic part of wine exhibits a protective effect on the lipid profile while phenolic compounds of wine decreased lipoprotein(a) plasma concentrations by 12%. Regarding lipoprotein(a), contradictory findings are also available in the literature [194].

It has been hypothesized that resveratrol is responsible for the cardioprotective effect of wine. Resveratrol protects against CVDs through lowering of lipid peroxidation, lowering of blood pressure, improvement of serum cholesterol profile, endothelial cells protection against apoptosis and reduction of platelet aggregation [195]. However, clinical findings are limited [196].

Virgin olive oil (VOO) is the primary fat source in the Mediterranean diet. It is rich in polyphenols, namely oleuropein, tyrosol and hydroxytyrosol and monounsaturated fatty acids (MUFA). Systolic blood pressure decreased after VOO intake in hypertensive patients, but diastolic blood pressure, glucose, lipids, and antibodies against oxidized LDL did not change. Its claimed that olive oil phenolics counteracted LDL oxidation due to both metals and radicals and acted as chain-breaking antioxidants for lipid peroxidation [197].

Aguilera et al. reported that sunflower-oil-enriched diets does not protect LDL against oxidation compared with VOO, since antioxidant activity of hydroxytyrosol and oleuropein found in VOO was more powerful than that of vitamin E found in sunflower oil. Furthermore, cardioprotective effect of MUFA rich diet resulted from an enhancement of HDL and a decrease in LDL levels [198].

Nuts (e.g., walnut, nut, pistachio, among others) contain unsaturated fatty acids, including omega-3 fatty acids and dietary fibre, which can lower the content blood cholesterol. Other essential bioactive nutrients are also found in nuts, such as vitamin E, potassium and magnesium, and non-nutrient bioactive compounds, such as ellagic acid, flavonoids, luteolin, and tocotrienols influence lipids and lipoproteins [199]. In a randomized crossover study [200], LDL-cholesterol decreased after consuming a diet enriched with VOO (7.3%), walnuts (10.8%) or almonds (13.4%). Besides, TC and LDL/HDL ratios decreased. Therefore, the authors concluded that diet enriched with nuts reduces cholesterol level and, consequently, exhibits beneficial properties on heart health.

Vegetables (e.g., beans, peas, chickpeas, and lentils) consumption is associated with lower CV disease risk, as they have a low glycemic index and low saturated fat content. Moreover, legumes are good sources of fiber, plant protein and potassium, each of which contributes to lowering blood pressure [201]. Legume proteins improve antioxidant response with decreasing oxidized LDLc levels [202]. A study [203] showed that non-soy legume rich diet decreased TC and LDLc. In another meta-analysis of a randomized controlled clinical trial (554 participants: overweight or obese, with diabetes, metabolic syndrome, without apparent disease), Jayalath et al. reported that a diet rich in legumes lead to a blood pressure lowering effect in predominantly middle-aged people with and without hypertension. The replacement of carbohydrates with protein led to reduced blood pressure. Also, the low glycemic index caused weight loss which contributes to blood pressure reduction. It was recommended an increase in consumption of at least two servings (1 cup) above current average intakes (0.1–0.3 servings/day) [201].

Whole-grain food consumption is also associated with lower CV disease risk. Whole-grain foods contain fibre, vitamins, minerals, phenolic compounds and phytoestrogens. These constituents have beneficial health properties: lower serum lipids and blood pressure, endothelial function improvement, alleviation of oxidative stress and inflammation [204].

In a clinical trial with 233 healthy middle-aged individuals, it was revealed that the daily consumption of three portions of whole-grain foods (wheat or a mixture of wheat and oats) significantly reduced the risk of CV disease in middle-aged, healthy, overweight men and women through blood pressure-lowering mechanisms [205]. The report stated that a decrease in systolic blood pressure could decrease the incidence of coronary artery disease (≥15%) and stroke (25%). Triglyceride, HDL, and apolipoprotein A1 concentrations did not change while total cholesterol (3.1%) and LDL-cholesterol (4.3%) concentrations decreased significantly. The hypocholesterolemic properties of viscous soluble fibre found in whole-grains contributed to their cardioprotective effect [205].

The encouraging results obtained from the epidemiological studies have generated substantial, randomized clinical trials with solid morbidity/mortality endpoints. A large number of meta-analyses have been published regarding the efficiency of polyunsaturated omega-3 fatty acids in the prevention of cardiovascular diseases, these meta-analyzes were generally positive and promoted the introduction of polyunsaturated fatty acids in primary prevention and in secondary prevention after myocardial infarction [206,207].

The Diet and Reinfarction Trial (DART) was the first secondary prevention study performed on post-myocardial infarction patients in the 1980s. The consumption of two fish meals per week led, after two years, to a total mortality decrease of 29% and a re-infarction of 32% [208].

The Gruppo Italiano per lo Studio della Sopravvivenza nell’Infarcto Miocardio (GISSI-Prevenzione) trial followed for 3.5 years after myocardial infarction over 11000 Italian patients who received 1 g daily of EPA + DHA, over the anti-aggregate therapy platelets (91%), conversion enzyme inhibitors (40%), β-blockers (40%), statins (45%). EPA + DHA managed to reduce cardiovascular mortality by 30%, coronary mortality by 35% and sudden death by 45%. The effect of fatty acid therapy occurred early (90 days for total mortality and 120 days for sudden death) and was four times stronger in patients with an ejection fraction below 40% [209].

GISSI Heart Failure, a large-scale, randomized, double-blind trial included over 7000 New York Heart Association NYHA II-IV heart failure patients treated with 1 g/day omega-3 fatty acids for 47 months, over standard disease therapy. Treatment with omega-3 fatty acids reduced cardiovascular mortality by 10%, sudden death by 7% and re-admission for ventricular arrhythmias by 28% [210]. The absolute risk reduction for all-cause mortality was 1.8%, omega-3 saving 18 lives per 1000 treated patients [210].

The Japan EPA Lipid Intervention Study (JELIS) was a prospective, randomized, open-label trial that included 18,645 hypercholesterolemic Japanese (20% with cardiovascular disease) who received for 5 years 1800 mg/day EPA and a low dose statin. Results of the study showed that the sudden cardiac death, myocardial infarction, unstable angina and revascularization procedures, was reduced by 19%, the reduction is achieved mainly on account of non-fatal events [211]. Fatal events (myocardial infarction and sudden death) were rare in JELIS, of only 0.2% per year, which can be explained by the existence of daily consumption of 1800 mg EPA + DHA, throughout this population. JELIS is a valuable study demonstrating the low incidence of fatal cardiovascular events in populations with an increased daily intake of polyunsaturated omega-3 fatty acids [211].

Although numerous older clinical studies have shown the positive cardioprotective effects of polyunsaturated fatty acids (PUFAs), there are also contemporary clinical trials that do not support their benefit.

Three studies were published in 2010: Alpha Omega, which included 4837 post-myocardial infarction patients, followed for 41 months (margarine supplementation), OMEGA, with 3851 post-IM patients, followed by 12 months (23) and SU.FOL.OM3 (SUpplementation with FOlate, vitamin B6 and B12 and/or OMega-3 fatty acids) performed on 2501 coronaries, for 56 months (24). In these studies, supplementation with 400–800 mg/day EPA + DHA did not reduce cardiovascular events [212].

Alpha-Omega trial was a multicenter, randomized, placebo-controlled study that divided the investigated cohort into four groups: ω3-PUFA + ALA, ω3-PUFA + ALA placebo, ω3-PUFA placebo + ALA, ω3-PUFA placebo + ALA placebo [213]. Inclusion criteria were between the ages of 60 and 80, a history of acute myocardial infarction not more than ten years ago and standard antithrombotic, hypolipidemic and antihypertensive treatment. Dietary supplementation with ω3-PUFAs did not reduce the incidence of major ischemic or non-ischemic cardiovascular events, sudden cardiac death or interventions such as angioplasty or aortocoronary bypass [213].

The OMEGA study included patients at least 18 years of age, but up to 70 years of age, with ST-elevation myocardial infarction( STEMI) or non-ST-elevation myocardial infarction (NSTEMI) within the first 3–14 days, treated according to good practice guidelines, excluding those with early revascularization, diabetes or left ventricular ejection fraction (LVEF), below 40%; subjects in the active arm received 1 g ω3-PUFAs with a DHA: PHA ratio of 380: 460 mg, and the placebo group 1 g of olive oil/day. Dietary supplementation with ω3-PUFAs has no additional beneficial effect compared to standard acute myocardial infarction therapy on overall mortality, the incidence of significant cardio- or cerebrovascular events, or sudden cardiac death [214]. It is worth mentioning that the design of the study was modified throughout to include patients at higher risk, but, with all post hoc changes, the research has low statistical power, and the short follow-up period (only one year) does that the long-term effects of polyunsaturated fatty acids should be neglected [214].

Supplementation with FOlate, vitamin B6 and B12 and/or OMega-3 fatty acids (SU.FOL.OM3) is a randomized, double-blind, placebo-controlled, 2 × 2 factorial study that investigated the effects of folic acid in combination with vitamin B6 or B12 and / or ω3-PUFAs in the secondary prevention of cardiovascular events (fatal or non-fatal). Participants with myocardial infarction, ischemic stroke, or unstable angina were randomized to receive the following supplements: ω3-PUFAs, ω3-PUFAs + 560 μg 5-MTHF, 3 mg vitamin B6, and 20 μg B12, vitamin B (3 mg vitamin B6 + 20 μg B12) and 560 μg 5-MTHF or placebo [215]. The results showed that vitamin B6 and/or B12, folic acid or ω3-PUFAs do not reduce cardiovascular risk in those with a history of ischemic heart disease or ischemic stroke [215].

Outcome Reduction with Initial Glargine Intervention (ORIGIN) is a randomized clinical trial which studied the effect of 1 g/day of ω3-PUFAs with a DHA: EPA ratio of 375: 465 mg in people at high risk of cardiovascular disease and diabetes, impaired glucose tolerance or impaired fasting blood glucose, did not show a statistically significant reduction in overall mortality, major cardiovascular events, or mortality due to arrhythmia [216]. The study participants were treated according to the standards, which may explain the lack of a beneficial effect, unlike other studies in which the cohort was not under standard treatment and which showed a positive impact. The study focused on a subgroup of individuals with diabetes, impaired glucose tolerance or impaired fasting blood glucose, which may influence the result. However, other clinical studies on the same subgroup have shown benefits [216].

Diet and Angina Randomized Trial (DART2) is a study which was intended to be the continuation of the 1989 DART study [208], with a similar design, focused this time on three aspects: i) investigating the deaths of participants with stable angina, in the DART study to distinguish between sudden cardiac death and other causes, in order to investigate the antiarrhythmic effect of polyunsaturated fatty acids; ii) focusing attention on another group of patients, men with stable angina and high risk for cardiovascular disease, as opposed to DART, in which participants had a history of myocardial infarction; thus, the role of PUFAs in different stages of ischemic heart disease is studied; iii) investigating the effects of increased consumption of fruits, vegetables and oats. The results of the study show that increased consumption of polyunsaturated fatty acid supplements not only is not beneficial but increases the number of cardiac deaths or sudden cardiac death, while the use of fruits, vegetables and oats has no positive effect (p 0.47 for cardiac death and p 0.98 for sudden cardiac death). Different conclusions compared to DART can be caused by the fact that in DART2 dietary supplements were used and not natural foods as sources of PUFAs, the compliance with the two types of diets was not identical, the participants being less adherent to the fruit-rich diet. and vegetables and the study groups were not standardized based on cardiovascular risk [208].

A Study of Cardiovascular EveNts in Diabetes (ASCEND) is the largest randomized controlled clinical trial of omega-3 fatty acid supplementation [217]. Between 2005 and 2011, 15,480 patients in the UK were randomly divided into two groups receiving either fish oil supplement (1 g per day) or placebo. The primary efficacy outcome was serious stroke, defined as non-fatal heart attacks, non-fatal strokes and deaths caused by a cardiovascular cause (except for intracranial haemorrhages) [218]. During the mean follow-up of 7.4 years, a first serious vascular event occurred in 689 (8.9%) participants assigned to fish oil supplements and 712 (9.2%) participants assigned to placebo. There was no significant difference between the two groups (relative risk of 0.97, 95% confidence interval 0.87–1.08, *p* = 0.55). There was also no significant difference in terms of mortality [218].

REDUction of Cardiovascular events with EPA-Intervention Trial (REDUCE-IT), a multicenter, randomized, double-blind study included 8179 patients with cardiovascular risk factors (diabetes) or cardiovascular disease, under statin treatment, with fasting triglycerides between 135–499 mg/dL and LDL cholesterol between 41–100 mg/dL. They were randomized, in a 1: 1 ratio, either in the twice-daily icosapent-ethyl treatment group or in the placebo group. The mean follow-up period of the study was 4.9 years. Icosapent-ethyl is an omega-3 derivative used to reduce serum triglycerides when values exceed 500 mg/dL. It has been associated in studies with other additional effects, such as anti-inflammatory and membrane stabilization, which may contribute to reducing cardiovascular risk [219].

The results of the REDUCE-IT study: patients with hypertriglyceridemia, despite statin treatment, given icosapent-ethyl 2g, recorded decreases in cardiovascular events compared to the placebo group major such as stroke or stroke (25%) and cardiovascular death (20%). In patients with another cardiovascular risk factor (diabetes mellitus), but without a major cardiovascular event, supplementation was not effective. It does not prevent heart attack or stroke [219].

VITAmin D and omegA-3 triaL (VITAL) is another interventional study with negative results in primary cardiovascular and cancer prevention. This is a randomized, placebo-controlled clinical trial which showed that the supplementation with omega-3 and vitamin D3 did not show a decrease in cardiac mortality [220]. During the 5.3-year follow-up of the study, omega-3 supplementation was associated with a statistically significant 28% decrease in the incidence of myocardial infarction (comparable to that of aspirin or statins in primary prevention), as well as reductions in fatal myocardial infarctions, percutaneous coronary interventions and total coronary heart disease. The participants with the most significant benefit were those with low fish intake, of less than 1.5 portions per week. There were no reductions in strokes, CV mortality, cancer incidence, cancer mortality or all-cause mortality [221].

Vitamin D3 supplementation decreased cancer mortality by 25% but did not significantly reduce cardiovascular events, total cancer incidence, or overall mortality. A subanalysis according to the body mass index, showed benefits of people with healthy weight. They registered significant decreases in cancer incidence and mortality of 24% and 25%, respectively, while obese or overweight people had no benefits. The VITAL study looked at the efficacy of omega-3 supplements (1 g per day) and with vitamin D3 (cholecalciferol, 2000 IU per day), in the primary CV and cancer prevention of 27,871 participants (men over 50 years and women over 55 years [221].

The negative results of the contemporary trials compared to the older clinical studies may be due to the fact that the patients included in the new studies received superior treatment, including statins and revascularization procedures, which were not available to the patients in the older studies [222]. The data of all these clinical trials are summarized in Table 3.

## 5. Conclusions and Future Perspectives

Dietary and healthy lifestyles are considered a practical approach to CV disease prevention. Analysis of in vitro, in vivo and clinical literature reports, indicate that the consumption of plant-food bioactive molecules belonging to a different class of natural compounds possesses cardioprotective effects on human health. In vivo studies offer the understanding of the mechanism of action, determine the bioactivity and interaction of the compounds within the body and synergistic and antagonistic effect. With increasing demand, natural health products can help to maintain and prevent cardiovascular diseases.

## Figures and Tables

**Table 1:** Benefits of Plant-derived Bioactive Constituents and Lifestyle Changes on Risk Factors in Cardiovascular Diseases.

Cardiovascular Risk Factor	Pathophysiology	Benefit	Ref
sedentary lifestyle	↑ insulin resistance, ↑ obesity, ↑blood glucose levels, ↑plasma lipids, ↑prothrombotic factors	lifestyle changes: physical activity lowers risk for CVDs mortality ↑physical activity, ↓ alcohol consumption, not smoking, ↓ obesity: ↓ risk of incident heart failure in 50% cases	[31] [32]
hypertension	↑arterial hypertension and endothelial growth factor-linked polymorphisms, ↑vascular damage	berberine, green tea, cocoa, lycopene, aged garlic extract, resveratrol, grape seed extract, beetroot juice, olive oil and ascorbic acid: ↓blood pressure. apricot and olive seed protein hydrolysates: ↓ACE Resveratrol: ↑ nitric oxid production, ↑vasodilatation	[36] [37]
smoking	↑oxidative stress, ↑coronary spasm, disorders of coagulation mechanisms, ↑platelet aggregation, fibrinogen growth, disorders of endothelial function ↑ LDLc, ↓ HDL	garlic extract, *Angelica gigas*, celery, *Artemisiae annuae* Herba, oat extracts, cinnamon, soy extracts, hibiscus, flaxseed, wolfberry, lycopene, basil, and cocoa ↓atherosclerosis proanthocyanins (bilberries): anti-inflammatory effect, ↓pro-inflammatory cytokines, ↓chemokines (TNFα, IL-1, IL-6, IL-8)	[49] [44]
stress	disturbance of the hypothalamo-pituitary-adrenal axis, ↑serum cortisol due to stress, ↑inflammation of the arterial (endothelial) wall, ↑atherosclerosis, ↑ tone of the sympathetic vegetative nervous system.	L-theanine from green tea: ↑relaxation, ↓post-stress cortisol hyperforin: ↑serotonin in brain, similar to antidepressant drugs ginseng: antistress action, ↓physical and mental fatigue, ↑ power of concentration, ↑memory, ↑work performance	[61] [62]
obesity	↑dyslipidemia, hypercoagulability, platelet dysfunction, ↑insulin resistance and type 2 diabetes, ↑inflammation	polyphenols (grapes, tea, ginseng, red to purple color fruits) benefits: ↓platelet aggregation, ↓vascular inflammation, ↓apoptotis, ↓LDL oxidation EPA and DHA: anti-inflammatory and cardioprotective effects carotenoids: ↑anti-oxidant potential resveratrol: ↓fat deposits, regulate insulin levels, ↓body weight, ↑SIRT 1 protein	[19] [79,80,81] [23]
diabetes	altering arterial walls increasing inflammation at the endothelial level, with the deposition of VLDL and LDL cholesterol particles at this level and the formation of atheroma plaque	mediterranean diet: ↓ risk of diabetes polyphenols, flavonoids, terpenoids, saponins and lignans: antihyperglycemic effect peptides (soybean): prevent T2D onset	[86] [110] [111]
dyslipidemia	increasing of atherogenic effect deposits of atheroma plaques on the internal walls of medium and large arteries, with thickening of the arterial wall and loss of elasticity	phenolic compounds, flavonols: ↓TC, ↓LDLc, ↓TG levels phytosterols: ↓LDLc Soybean components, both proteins and isoflavones: ↓LDLc, ↑HDLc	[119] [120] [121] [127] [128]

*Abbreviations*: cardiovascular diseases (CVDs), total cholesterol (TC), low-density lipoprotein cholesterol (LDLc), high-density lipoprotein cholesterol (HDLc), type II diabetes (T2D), interleukin (IL), tumor necrosis factor alpha (TNFα), Angiotensin-converting enzyme (ACE).

**Table 3:** Clinical Studies Related to Cardioprotective Effects of Plant-food Bioactive Compounds.

Plant-Food Bioactive Compounds	Clinical Study	Results	Ref
polyphenols (anthocyanins)	observational study on three groups of patients with CVDs consuming mediterranean diet	↑endothelial function and plasma lipid profiles ↓abnormal platelet aggregation, ↓oxidative stress	[185] [186] [187] [189]
pomegranate juice (anthocyanins, catechins, quercetin, rutin, ellagitannins)	single-blind study for hypertensive men	↓blood pressure due to its ACE activity antioxidant activity: due to the radical scavenging effect of anthocyanins and hydrolysable tannins	[190]
cranberry juice (anthocyanins, polyphenols)	a placebo-controlled crossover study in patients with coronary artery disease	↓carotid femoral pulse wave velocity only acute benefit but not a chronic effect on endothelial vasodilator function	[132] [191]
anthocyanins	a clinical study in hypertensive patients	anthocyanins did not exhibit anti-hypertensive activity in borderline hypertensive men	[132]
blueberries	a double-blind, placebo-controlled study in subjects with metabolic syndrome	blueberries did not affect blood pressure and insulin sensitivity ↑endothelial function	[184]
red wine (resveratrol)	randomized clinical trial	the ethanolic part of wine: ↑protective effect on the lipid profile phenolic compounds of wine: ↓lipoprotein plasma concentrations by 12%, ↓lipid peroxidation, ↓ blood pressure, ↓ serum cholesterol, ↓platelet aggregation endothelial cells protection against apoptosis	[194] [193] [195] [196]
virgin olive oil (oleuropein, tyrosol, hydroxytyrosol, monounsaturated fatty acids)	randomized, crossover controlled clinical trial in hypertensive patients	↓ systolic blood pressure, ↓ diastolic blood pressure, glucose, ↓lipids antibodies against oxidized LDL did not change olive oil phenolics counteracted LDL oxidation due to both metals and radicals and acted as chain-breaking antioxidants for lipid peroxidation ↑HDL, ↓LDL	[174]
sunflower-oil (vitamin E)	clinical study of Spanish male patients with peripheral vascular disease	sunflower-oil-enriched diets didn’t protect LDL against oxidation	[198]
nuts (unsaturated omega-3 fatty acids, dietary fibers, vitamin E, potassium and magnesium)	randomized crossover study	↓ blood cholesterol ↓ LDL-cholesterol ↓TC, ↓LDL/HDL ratios	[199] [200]
vegetables (fibre, plant protein, K)	randomized controlled clinical trial in patients with diabetes, metabolic syndrome without apparent disease	↑antioxidant response, ↓oxidized LDLc ↓blood pressure, ↓TC, ↓LDLc	[201] [202] [203]
whole-grain food (fibre, vitamins, minerals, phenolic compounds, phytoestrogens)	a clinical trial with 233 middle-aged, healthy, overweight men and women	↓serum lipids, ↓blood pressure, ↑endothelial function, ↓oxidative stress, ↓inflammation, ↓systolic blood pressure triglycerides, HDL, and apolipoprotein A1 concentrations did not change ↓ total cholesterol, ↓LDL-cholesterol	[205] [204]
unsaturated omega-3 fatty acids	secondary prevention trial performed on post-myocardial infarction patients (DART)	total CV mortality ↓of 29% and a re-infarction of 32%.	[208]
	preventive clinical trial patients after myocardial infarction (GISSI-Prevenzione trial)	EPA + DHA reduced cardiovascular mortality by 30%, coronary mortality by 35% and sudden death by 45%.; the effects occurred early (90 days for total mortality and 120 days for sudden death) and were 4 times stronger in patients with an ejection fraction below 40%	[209]
	large-scale, randomized, double-blind clinical study (GISSI Heart Failure)	omega-3 fatty acids reduced cardiovascular mortality by 10%, sudden death by 7% and re-admission for ventricular arrhythmias by 28%	[210]
	hypercholesterolemic patients prospective, randomized, open-label study (JELIS)	the sudden cardiac death, myocardial infarction, unstable angina and revascularization procedures, were reduced by 19%	[211]
	a multicenter, randomized, placebo-controlled study in post-myocardial infarction patients (Alpha Omega)	dietary supplementation with ω3-PUFAs did not reduce the incidence of major ischemic or non-ischemic cardiovascular events, sudden cardiac death or interventions such as angioplasty or aortocoronary bypass	[213]
	randomized, placebo-controlled, double-blind, multicenter trial included patients after myocardial infarction (OMEGA)	dietary supplementation with ω3-PUFAs has no additional beneficial effect compared to standard acute myocardial infarction therapy on overall mortality, the incidence of major cardio- or cerebrovascular events, or sudden cardiac death.	[214]
	randomized double-blind, placebo-controlled secondary-prevention trial in patients with myocardial infarction, ischemic stroke, or unstable angina (SU.FOL.OM3)	no reduction of cardiovascular risks in patients with a history of coronary heart disease or ischemic stroke.	[215]
	randomized clinical trial in people at high risk of cardio- vascular disease and diabetes, impaired glucose tolerance or impaired fasting blood glucose (ORIGIN)	the study did not show a statistically significant reduction in overall mortality, major cardiovascular events, or mortality due to arrhythmia.	[216]
	randomized trial in participants with stable angina (DART2)	increased consumption of polyunsaturated fatty acid supplements was not beneficial in reducing cardiac deaths or sudden cardiac death, the use of fruits, vegetables and oats has no positive effect on cardiovascular risk factors	[208]
	randomized controlled clinical trial in patients with diabetes, but without a history of cardiovascular disease (ASCEND)	the study did not show a decrease in mortality	[218]
	a multicenter, randomized, double-blind controlled trial in patients with cardiovascular risk factors (diabetes) or cardiovascular disease (REDUCE-IT)	patients with hypertriglyceridemia recorded decreases in cardiovascular events compared to the placebo group major such as stroke or stroke (25%) and cardiovascular death (20%) in patients with another cardiovascular risk factor (diabetes mellitus), but without a major cardiovascular event, supplementation was not effective. It does not prevent heart attack or stroke	[219]
	a randomized, placebo-controlled trial study (VITAL)	no reductions in strokes, CVDs mortality, cancer incidence, cancer mortality or all- cause mortality	[220] [221]

*Acronymss, abbreviations and symbols*: ↑ (increase), ↓ (decrease), CV (cardiovascular), CVDs (cardiovascular diseases), Angiotensin-Converting Enzyme (ACE), DART (Diet and Reinfarction Trial), GISSI (Gruppo Italiano per lo Studio della Sopravvivenza nell´Infarcto Miocardio), JELIS (Japan EPA Lipid Intervention Study), SU.FOL.OM3 (SUpplementation with FOlate, vitamin B6 and B12 and/or OMega-3 fatty acids), DART 2 (Diet and Angina Randomized Trial), ASCEND (A Study of Cardiovascular Events in Diabetes), REDUCE-IT (Reduction of Cardiovascular events with EPA - Intervention Trial), VITAL (VITamin D and OmegA-3 TriaL).
